# Can treating critically-ill haematological malignancy patients in a separate intensive care unit decrease intensive care unit mortality?

**DOI:** 10.3906/sag-2012-107

**Published:** 2021-08-30

**Authors:** Gülbin AYGENCEL, Nazlıhan BOYACI DÜNDAR, Melda TÜRKOĞLU, Zeynep Arzu YEGİN, Zübeyde Nur ÖZKURT, Abdullah Münci YAĞCI

**Affiliations:** 1 Division of Intensive Care Medicine,Department of Internal Medicine, Gazi University Faculty of Medicine, Ankara Turkey; 2 Division of Hematology,Department of Internal Medicine, Gazi University Faculty of Medicine, Ankara Turkey

**Keywords:** Intensive care unit, separate intensive care unit, patients with haematological malignancies, intensive care unit mortality

## Abstract

**Background/aim:**

The aim of the study was to investigate whether treating haematological malignancy (HM) patients in a separate intensive care unit (ICU) would reduce ICU mortality.

**Materials and methods:**

HM patients treated by the same ICU team in a general medical ICU (GM-ICU) and a separate haematology ICU (H-ICU) were included in this study. Patients’ demographic characteristics and ICU data were recorded retrospectively. Differences in the ICU course and prognosis between these two groups were determined.

**Results:**

A total of 251 patients (102 from GM-ICU, 149 from H-ICU) were included in this study. The disease severity and organ failure scores at ICU admission and underlying HMs were not different between the two groups. Patients waited longer for admission to GM-ICU. Therapeutic procedures were performed significantly more frequently in GM-ICU. ICU complications were not different between the groups. ICU mortality rates were higher in GM-ICU (59.8% vs 37.6%, p = 0.006).

**Conclusion:**

A separate ICU allocated for haematology patients will allow timely and rapid admission of HM patients to ICU. Thus, mortality rates of HM patients needing ICU care will decline.

## 1. Introduction

Intensive care units (ICUs) should have a rational triage system for patient admission because of the limited ICU bed capacity. The high ICU mortality rate and ambiguity concerning the effects of life or organ support in critical cancer patients, especially patients with haematological malignancies (HMs), lead to the development of passive resistance to admitting such patients in ICUs [1–3]. 

The presence of a separate ICU for critically-ill cancer patients, especially critically-ill HM patients, eliminates the triage steps for the admission of such patients; ensuring avoiding the competition for the same bed across patients with different diseases and enables critically-ill HM patients to get ICU support timely and rapidly. Implementation of a separate ICU for critically-ill cancer patients, especially critically-ill HM patients will contribute to the accumulation of knowledge and experience further in this special patient group and promote early interventions for some specific situations that might remain unnoticed in a general ICU resulting in decreases in morbidity and mortality.

In this study, we compared HM patients treated in a separate haematology ICU (H-ICU) with HM patients treated in a general medical ICU (GM-ICU) to show whether there were differences in the ICU course and mortality between these two groups of patients during the ICU stay. 

## 2. Materials and methods

Gazi University Hospital is a tertiary referral hospital with approximately 1000 beds in the city of Ankara. In the hospital, there is a 35-bed haematology clinic and an 8-bed bone marrow transplant unit. Because of delays in admissions to GM-ICU, a 4-bed tertiary ICU (H-ICU) was established in the hospital in 2014 for critically-ill haematology patients. Another aim to establish a separate H-ICU was to avoid treating such immunocompromised patients in the same unit where patients with other types of diseases are treated, too. The organizational and administrative tasks of the newly established H-ICU were assigned to the ICU team experienced in the follow-up and treatment of HM patients in the GM-ICU. Currently, an internal medicine physician is available in the H-ICU for 7 days and 24 h, and an ICU specialist is available during working hours. In the newly established H-ICU, 100-120 patients receive treatment in a year. Patients admitted to H-ICU are usually composed of HM patients. 

We planned this retrospective study to determine whether treating critically-ill HM patients in a separate H-ICU acted on ICU mortality rates. The study included HM patients treated in the GM-ICU within the 2 years (in the period between January 01, 2012 and December 31, 2013) before the establishment of H-ICU and included HM patients treated in the H-ICU within the 2 years after its establishment (in the period between January 01, 2014 and December 31, 2015). Patients who stayed in the ICU longer than 24 h and first admission of the patients who were admitted to the ICU more than once were included in this study. In addition to the demographic characteristics of the patients, the following data for each patient were recorded including the characteristics referral information for the ICU admission (from where, when, why, etc), co-morbidities, type of HM, disease status (new diagnosis, under control, relapse, end-stage etc.), vital signs at admission, acute disease severity and organ failure scores (acute physiology and chronic health evaluation - APACHE II score, sequential organ failure assessment - SOFA score, Glasgow coma scale - GCS etc. ) at admission, laboratory values at admission, therapeutic procedures that the patient underwent during the ICU stay (mechanical ventilation – MV, renal replacement therapy – RRT, etc.), existing infections at ICU admission, infections and complications (gastrointestinal - GI bleeding, sepsis, acute kidney injury – AKI, etc.) during the ICU stay, and ICU outcomes (survival or death ). Then, the data of critically-ill HM patients treated in GM-ICU were compared with those of critically-ill HM patients treated in H-ICU to find out whether there were differences between these two groups, especially in terms of ICU course and outcomes.

### 2.1. Statistical analysis

Statistical analysis was performed using the IBM SPSS (Statistical Package for Social Sciences) statistical software package version 22.0 (IBM Corp., Armonk, NY, USA). Continuous variables were reported as mean ± standard deviations or median [interquartile ranges]. Frequencies and percentages were used for the presentation of categorical variables. Patients were divided into two groups as HM patients treated in GM-ICU and HM patients treated in H-ICU. The Mann–Whitney U test or Student’s t-test was used to compare continuous variables, the chi-square test or Fisher’s exact test was used to compare categorical variables. P values lower than 0.05 were considered statistically significant.

## 3. Results

A total of 251 patients were included in the study. The numbers of critically-ill HM patients treated in GM-ICU and H-ICU were 102 and 149, respectively. No statistically significant differences were observed between the two groups in APACHE II, SOFA, and GCS scores at ICU admission, the length of ICU stay, sex distribution, underlying haematological malignancies, disease status, the status and type of haematopoetic stem cell transplantation (HSCT) (Table 1). While patients admitted to H-ICU were older, end-stage cancer and comorbidities in this patient group were more frequent; patients admitted to GM-ICU waited longer for ICU admission and suffered from respiratory failure more frequently at the time of ICU admission (Table 1).

**Table 1:** General characteristics of all study patients, and patients in haematology ICU and general medical ICU.

Parameter	All study patients(n=251)	Patients in haematology ICU (n=149)	Patients in general medical ICU (n=102)	P Value
Age*	58 [47-66]	59 [49.5-66.5]	56 [42.75-64.25]	0.035
Admission	23 [19-29]	23 [19-28.5]	23 [18-29]	0.78
APACHE II score*				
Admission GCS *	14 [9-15]	14 [9-15]	13 [9-15]	0.212
Admission	8 [6-12]	8 [5-11]	9 [6-12]	0.352
SOFA score*				
Length of ICU stay*	6 [3-13]	6 [3-15]	6.5 [4-12]	0.969
(days)				
Waiting time for ICU	8 [4-16]	5 [3-9]	16 [8-22.5]	0.0001
admission* (hours)				
Sex, M, n (%)	161 (63.9)	94 (63.1)	67 (65.7)	0.673
Underlying hematologic malignancies, n (%)				
Acute Leukemia	113 (44.8)	68 (45.6)	45 (44.1)	0.812
Multiple Myeloma	76 (30.2)	47 (31.5)	29 (28.4)	0.598
Lymphoma	68 (27)	37 (24.8)	31 (30.4)	0.330
Status of hematological malignancy, n (%)				
Recently-diagnosed	88 (34.9)	48 (32.2)	40 (39.2)	0.659
Relapsed	94 (37.3)	52 (34.9)	42 (41.2)	0.313
In remission	40 (15.9)	25 (16.8)	15 (14.7)	0.659
End-stage	21 (8.3)	17 (11.4)	4 (3.9)	0.035
HSCT, n (%)				
Allogeneic	54 (21.4)	28 (18.8)	26 (25.5)	0.205
Autologous	45 (17.9)	28 (18.8)	17 (16.7)	0.666
Co-morbidities, n (%)				
Diabetes Mellitus	56 (22.2)	44 (29.5)	12 (11.8)	0.001
Chronic heart diseases	53 (21)	40 (26.8)	13 (12.7)	0.007
Chronic renal diseases	47 (18.7)	31 (20.8)	16 (15.7)	0.307
Chronic lung diseases	28 (11.1)	20 (13.4)	8 (7.8)	0.168
Reasons for ICU admission, n (%)				
Sepsis/septic shock	192 (76.2)	111 (74.5)	81 (79.4)	0.367
Respiratory failure	180 (71.4)	99 (66.4)	81 (79.4)	0.025
Renal failure	78 (31)	40 (26.8)	38 (37.3)	0.08
Change in	62 (24.6)	39 (26.2)	23 (22.5)	0.513
consciousness				

* median [interquartile ranges], n: numberICU: intensive care unit, APACHE: acute physiology and chronic health evaluation, GCS: Glasgow coma scale, SOFA: sequential organ failure assessment, M: male, HSCT: hematopoietic stem cell transplantation.

There were no differences in vital signs between the two groups at ICU admission but haemoglobin, procalcitonin, sodium, and LDH levels were significantly different between the two groups at the time of ICU admission (Table 2). Pulmonary sepsis as a cause of ICU admission was more common in patients admitted to GM-ICU (Table 2). Invasive mechanical ventilation support was more common in GM-ICU patients at ICU admission. As for diagnostic and therapeutic procedures performed in ICU, invasive mechanical ventilation (IMV) support and arterial catheterization were significantly more commonly performed in GM-ICU (Table 2). The development of complications (GI bleeding, AKI, arrhythmias, nosocomial infections, etc.) during the ICU stay were not different between the groups (Table 2). The comparison of the groups for mortality in ICU revealed that 63.7% of the patients treated in GM-ICU died, and 49% of the patients treated in H-ICU died. The difference between the groups was statistically significant (p = 0.021) (Table 2). When end-stage HM patients were excluded from both groups, the net ICU mortality rate was 59.8% in GM-ICU patients, and 37.6% in H-ICU patients. The difference in ICU mortality rates was statistically significant between the two groups (p = 0.006) (Table 2).

**Table 2:** ICU admission and follow-up characteristics of all study, haematology ICU and general medical ICU patients ​included in the study.

Parameter	All study patients(n=251)	Patients in haematology ICU (n=149)	Patients in general medical ICU (n=102)	P
Vital signs at ICU admission *				
Body temperature (°C)	36.7 [36.4-37.1]	36.7 [36.4-37]	36.7 [36.3-37.28]	0.995
Pulse ( /min)	119 [102.5-131]	119 [104-132]	117 [102-130]	0.714
Mean arterial pressure	72 [62-84.5]	71 [62-84]	74 [62.25-85.75]	0.256
(mmHg)				
Respiratory rate (/min)	28 [23-32]	28 [24-34]	26 [22-32]	0.136
Some laboratory parameters at ICU admission				
Hemoglobin* (g/dL)	8 [7.18-9.2]	7.8 [6.7-8.88]	8.47 [7.40-9.7]	0.002
White blood cell*	4400 [800-	3994 [515-9104]	4775 [1510-	0.128
( /mm 3)	10490]		11315]	
Neutropenia, n (%)	98 (38.9)	63 (42.3)	35 (34.3)	0.398
Procalcitonin*	2.95 [0.7-19.8]	1.89 [0.4-9.64]	6.74 [1.85-31]	0.0001
(ng/mL)				
Creatinine* (mg/dL)	1.15 [0.67-2.22]	1.09 [0.6-1.86]	1.19 [0.7-2.44]	0.146
Sodium* (mEq/L)	137 [133-141]	136 [133-140]	138 [135-143]	0.05
ALT* (U/L)	20 [12-39]	20 [12-42]	21 [12-36.25]	0.771
LDH* (U/L)	378 [268-720]	353 [245-672]	472.5 [312.3-859.8]	0.006
Albumin* (g/dL)	2.6 [2.2-3]	2.63 [2.2-3.03]	2.6 [2.3-3]	0.885
Origin of sepsis at ICU admission, n (%)				
Pulmonary	156 (61.9)	84 (56.4)	72 (70.6)	0.023
Bloodstream/catheter	44 (17.5)	27 (18.1)	17 (16.7)	0.766
related bloodstream				
Abdominal	34 (13.5)	17 (11.4)	17 (16.7)	0.232
Urinary	24 (9.5)	10 (6.7)	14 (13.7)	0.063
IMV support at ICU	63 (25)	30 (20.1)	33 (32.4)	0.045
admission, n (%)				
Vasopressor support at	84 (33.3)	47 (31.5)	37 (36.3)	0.372
ICU admission, n (%)				
Procedures performed during ICU stay, n (%)				
NIV	112 (44.4)	63 (42.3)	49 (48)	0.367
IMV	161 (63.9)	86 (57.7)	75 (73.5)	0.01
Arterial	193 (76.6)	104 (69.8)	89 (87.3)	0.001
catheterization				
Central venous	189 (75)	107 (71.8)	82 (80.4)	0.122
catheterization				
RRT	65 (25.8)	42 (28.2)	23 (22.5)	0.317
Complications developed during ICU stay, n (%)				
Nosocomial infections	80 (31.7)	51 (34.2)	29 (28.4)	0.333
AKI	83 (32.9)	45 (30.2)	38 (37.3)	0.243
Cardiac	25 (9.9)	17 (11.4)	8 (7.8)	0.354
GI bleeding	22 (8.7)	15 (10.1)	7 (6.9)	0.378
Pneumothorax	11 (4.4)	8 (5.4)	3 (2.9)	0.533
Crude ICU mortality	138 (54.8)	73 (49)	65 (63.7)	0.021
rate, n (%)				
Net ICU mortality rate,	117 (46.6)	56 (37.6)	61 (59.8)	0.006
n (%) **				

* median [interquartile range], n: number,

## 4. Discussion

Haematological malignancy patients require frequently ICU admissions due to comorbidities, primary disease, and treatment-associated side effects. But for years, the ICU admission of HM patients has created an ethical dilemma because of poor prognosis and high mortality rates in this patient group during their stay in ICU. Despite the developments in cancer treatment and organ support therapies enabling this patient group to achieve better prognostic outcomes over the last 20–30 years [1–4], ICU mortality is still high (30%–80%) [2,5–8]. In this retrospective study, we showed that outcome and prognosis of HM patients who required ICU care were better if treated in its own ICU as in new study by Kalicińska et al [8]. We found that ICU mortality rate of HM patients treated in a separate and private ICU, namely H-ICU, was significantly lower than that of HM patients treated in GM-ICU in spite of similar haematological disease characteristics, disease severity and organ failure scores (49% vs 63.7%, p = 0.021). Moreover, when one considered the higher number of end-stage HM patients in H-ICU, the difference between mortality rates of GM-ICU and H-ICU became even more striking (37.6% vs 59.8%, p = 0.006).

As HM patients are considered to have a poor prognosis, ICU admission is not prioritized for these patients in triage system. However, instead of considering all these patients as the same, it may be more prudent to perform a customized evaluation for each patient. In some studies, it has been reported that some factors at ICU admission or during the ICU stay determine the ICU prognosis. These factors include type and the status of HM at ICU admission, age, presence or absence of alternative treatment options for HM, neutropenia, presence and type of HSCT procedure, graft versus host disease (GVHD), severity of the acute disease, requirement of IMV support, need of vasopressor and inotrope for hemodynamic stability, presence of sepsis, invasive fungal infections, severe comorbidities and multiple organ failures, and need of organ support therapies [9–18]. Determining the factors affecting the prognosis of HM patients was beyond the scope of this study. However, we observed that the ICU mortality rate was high in HM patients who experienced a long waiting time until ICU admission, who were admitted to ICU due to respiratory failure and pulmonary sepsis, who had higher procalcitonin, sodium, and lactate dehydrogenase levels at the ICU admission, and who required more invasive arterial monitoring and IMV support during the ICU stay. All of these findings suggested that the admission of HM patients to our GM-ICU was delayed. The increase in survival of HM patients who need ICU care, can only be achieved by eliminating the prejudice existing for these patients in the ICU triage system or by establishing special ICUs for these patients. 

Early detection of haemodynamic and respiratory deteriorations and rapid initiation of necessary treatments before the development of multiple organ failures are important for prognosis in HM patients [11–13]. Therefore, patients need to be transferred to ICUs swiftly. In ICUs accepting patients, regardless of the diagnosis, early ICU admission of HM patients is often hardly possible because of the high number of patients on the waiting list and because HM patients are not prioritized in the ICU triage system. Indeed, our patients, too, were admitted to our GM-ICU after a long waiting period. This delay may explain the high mortality rate in this patient group in our study. Again, HM patients were admitted to our GM-ICU mostly due to respiratory failure and they needed IMV support more. However, if these patients had been admitted to the ICU earlier, they could have received early non-invasive mechanical ventilation (NIV) support or high flow nasal oxygen (HFNO) therapy rather than receiving IMV support, and they could have recovered better and mortality could have been lower. Achieving such favourable outcomes can only be made possible through the establishment of specialized ICUs admitting only these patients. The establishment of specialized ICUs for these patients may allow to find the chance for early admission not only for the treatment of respiratory failure but also for the monitorization of hemodynamic parameters and for the management of metabolic disorders and sepsis. 

It is known that prognosis is better in cancer patients treated in a specialized ICU or a specialized centre compared to cancer patients treated in a general ICU or a centre admitting patients with any diagnosis. Specialization of a unit or centre results in monitoring and treating a large number of patients having the same diagnosis, leading to accumulating experience and knowledge on the specialized subject. This, in turn, will enable the utilization of specialized experience and knowledge in the treatment of patients [19]. This is a subject matter, which has been previously proven by Kahn et al. and by Shahin et al. in studies on mechanically ventilated patients [20,21]. Again, monitoring a large number of patients with a specific diagnosis can enable to establish a better organizational structure, develop clearer protocols, build multidisciplinary teams, and perform better staffing in a given centre. Reduced mortality in the presence of an increased number of cases (case-volume) was demonstrated previously in haematological patients by Lecuyer et al. and by Hampshire et al. [19,22].

In our study, end-stage HM patients were more frequent in the H-ICU. This may be due to two reasons. The first one is that the haematologist and ICU specialist could not reach a consensus on the prognosis of the patient and they considered to make a decision by following the patient in ICU. The second one is that H-ICU has been established as a specialized unit to treat only HM patients, but it turned out to serve as a palliative care unit, too. However, in the latter case, it may be hard to benefit from H-ICU for both purposes because of the inadequate bed capacity and the team’s lack of knowledge on palliative care. 

Our study has some limitations. Firstly, our study is retrospective. There may be data loss in retrospective studies. Secondly, it is a single center study meaning that the results of the study cannot be generalized because of the use of local protocols and approaches for ICU admission, discharges, procedures in patient care, and treatment. Thirdly, it is necessary to demonstrate long-term results and the quality of life after patients are discharged from H-ICU. Lastly, it is required to determine the cost-effectivity of the establishment of this specialized H-ICU and giving patient care in such a unit.

In conclusion, the availability of a separate haematology intensive care unit enabled haematological malignancy patients to have access to intensive care in a timely manner. This decreased the ICU mortality rates of patients with haematological malignancies. However, multicentre, large-scale studies are needed to confirm our results and demonstrate the effects of such specialized units on long-term survival and the quality of life. 

## Informed consent

This study was approved by the Ethics Committee of Keçiören Training and Research Hospital (date: June 24, 2015 and number: 886).
